# Applying a Socio-Ecological Model to Understanding the Needs of Children and Young People Bereaved by Intimate Partner Homicide across their Life Course

**DOI:** 10.1007/s10896-024-00721-z

**Published:** 2024-07-29

**Authors:** Zain Kurdi, John Devaney, Claire Houghton, Oliver Eastwood, John Frederick, Kathryn Joy, Katitza Marinkovic Chavez, Ashwini Sakthiakumaran, Eva Alisic

**Affiliations:** 1 School of Social and Political Science, University of Edinburgh, 15a George Square, Edinburgh, EH8 9LD UK; 2 Melbourne School of Population and Global Health, The University of Melbourne, 207 Bouverie St, Carlton, VIC 3053 Australia; 3 Department of Social Work, Monash University, Melbourne, Australia

**Keywords:** Family violence, Traumatic grief, Lived experience, Children & Adolescents, Trauma informed care, Children’s rights, Intimate partner homicide

## Abstract

**Purpose:**

To develop a socio-ecological understanding of the immediate and long-term effects on, and the needs of, children and young people (CYP) in the UK and Ireland bereaved by parental intimate partner homicide (IPH).

**Method:**

The study draws on in-depth interviews from three different informants: those with lived experience (LE) (10); caregivers (12); and professionals (10). In addition to the 10 interviews with those bereaved by maternal IPH, experiences discussed include a further 23 cases of bereavement due to IPH during childhood (when aged under 18). We used thematic analysis to code and extracted themes into the relevant five dimensions of the socio-ecological model.

**Results:**

The circumstances in which the homicide/suicide took place, were crucial in shaping children’s life trajectory. We identified circumstantial predictors in branching of trajectories: witnessing the murder; relatedness to perpetrator; and assumptions on biological relatedness. We found the need for life-long access to therapeutic care to support CYP essential during various developmental stages and life transitions. We recognized that both kinship and foster carers, required support in dealing with the complexity of grieving children. For kinship care, carers require help in managing their own grief, in addition to financial support to account for the new caring responsibilities. We also observed that children’s voice was rarely elicited, with no opportunities to exercise their agency.

**Conclusion:**

Our findings highlight the importance of contextual circumstances for tailored support and the identification of appropriate carers and supporting them holistically. Finally, we highlight the importance of child centered policies and dedicated resources to support relevant services dealing with CYP bereaved by parental IPH.

## Introduction

There is limited primary research exploring the immediate and longer-term trauma and needs of children and young people (CYP) bereaved by parental intimate partner homicide (IPH) in the UK and internationally. This scarcity in evidence, alongside other factors, has meant that for children who survive IPH, safeguarding policies aimed at ensuring the immediate care of the child(ren) are not geared towards the intricate nature and dynamics of bereavement due to parental IPH. Professionals across services have to make profound decisions concerning guardianship, placement, counselling and mental health, as well as potential contact with the accused (who is often the child’s other biological parent) without a strong evidence base to support these decisions (Alisic et al., 2015).

A systematic review on the global prevalence of IPH concluded that 1 in 7 homicides globally and more than a third of femicides are committed by an intimate partner (Stöckl et al., 2013). The world report on violence and health stated that internationally, an estimated 40%-70% of murdered women are killed by a current or former partner (Krug et al., 2002). This translated to 13.5% of homicides globally being committed by a current or former partner with women experiencing this at a rate six times higher than men (Stöckl et al., 2013). IPH is no doubt a gendered phenomenon worldwide; in the UK the Office for National Statistics confirms the vast majority of domestic homicide victims are women (2023), the latest femicide census reports a total of 110 women were killed, 57 by a current or former partner (Allen et al., 2022). There is little data on children in these circumstances, though a Netherlands study aiming to measure the prevalence of children bereaved by IPH over the period 2003-2013 (16.8 million population in 2013), identified 256 children that lost a biological parent due to IPH (Alisic, Groot, Snetselaar, Stroeken, Hehenkamp, & van de Putte, 2017; Alisic, Groot, Snetselaar, Stroeken, & van de Putte, 2017). The global review acknowledges that these homicides often represent the culmination of a long history of abuse and the human cost of IPH impacts surviving children who experience significant changes to their social environments (Stöckl et al., 2013).

Reviews of the literature on children bereaved by IPH identify children as the neglected victims – both in research and in practice responses (Alisic et al., 2015; Mertin, 2019). Clinical case records, descriptive data and proxy informants (e.g. Black et al., 1992; Burman & Allen-Meares, 1994; Lewandowski et al., 2004) reveal much about children’s circumstances as well as mental health symptoms connected with trauma and grief. This scenario may involve one parent’s death, while the other parent is either incarcerated, fleeing, or has died by suicide, often resulting in the family home becoming a crime scene (Black et al., 1992; Burman & Allen-Meares, 1994). Unfortunately, in numerous cases, children lose their mother due to the actions of their father (Alisic, Groot, Snetselaar, Stroeken, Hehenkamp, & van de Putte, 2017; Alisic, Groot, Snetselaar, Stroeken, & van de Putte, 2017; Allen et al., 2022). Many studies reveal that children often witness the killing or find the body, which increases the likelihood of moderate to severe PTSD, all children bereaved through IPH experience trauma symptoms, multiple losses and severe disruption to their lives (Alisic et al., 2015; Mertin, 2019). There is a need for mental health services immediately after the death occurs, assessment of PTSD, expert advice on access, the body, the funeral and immediate support for all involved in placement (Black et al., 1992) including therapeutic strategies for grief, anxiety, family communication and interaction (Burman & Allen-Meares, 1994). Yet few receive appropriate ongoing psychiatric care, or indeed appropriate intervention from any professional (Alisic et al., 2015; Lewandowski et al., 2004; Mertin, 2019).

More recently (with rare early exceptions e.g. Eth & Pynoos, 1994) primary research with children or adults bereaved as children (Alisic, Groot, Snetselaar, Stroeken, Hehenkamp, & van de Putte, 2017; Alisic, Groot, Snetselaar, Stroeken, & van de Putte, 2017; Steeves et al., 2011), caregivers (Enander et al., 2024; Hardesty et al., 2008) and professionals (Gomersall et al., 2024) is deepening our understanding of the experiences, perspectives and ongoing needs of those bereaved through IPH. Research undertaken with 23 children and young people (aged 8 – 24) bereaved due to parental IPH before the age of 18 in the Netherlands found that they faced persistent difficulties in the aftermath of bereavement (Alisic, Groot, Snetselaar, Stroeken, Hehenkamp, & van de Putte, 2017; Alisic, Groot, Snetselaar, Stroeken, & van de Putte, 2017). These included distress, conflict between family members and feelings of unsafety (Alisic, Groot, Snetselaar, Stroeken, Hehenkamp, & van de Putte, 2017; Alisic, Groot, Snetselaar, Stroeken, & van de Putte, 2017). Steeves and Parker (2007) asked what helped or hindered 47 adult survivors in the aftermath of parental homicide and they spoke about suffering and meaning making; the need to discover as much as possible, assign reasoning, to seek counselling at different times/triggers, make peace and have a strong figure in their life. Steeves et al., 2011 focus on adolescent’s experiences revealed post placement abuse, trauma exacerbated by media and professionals, varied experiences and differing narratives and outcomes (both positive and negative) of isolating or integrating the death into their adult lives. Research with 22 bereaved family members, including adult children of IPH victims, focused on their poor experiences of information and support following IPH (Enander et al., 2024) which contributed to negative mental health consequences. They recounted that social support following IPH was lacking or inadequate, that they had been left alone to handle difficult tasks, citing immediate and ongoing gaps in institutional response regarding information, understanding and coordination, continuity and professionalism (Enander et al., 2024).

These studies all highlight children’s need to talk and constraining circumstances and people, an example is boys in Steeves et al. (2007) who thought avoiding talking was unhelpful and the reactions of others were a main reason for the harmful, dangerous and persistent silence that ensued; they expressed the need for someone to listen who will not be hurt or distressed and a choice whether, when and who to talk to. Alisic, Groot, Snetselaar, Stroeken, Hehenkamp, & van de Putte, 2017; Alisic, Groot, Snetselaar, Stroeken, & van de Putte, 2017 study points us towards children’s competence, wishes and needs: that professionals and others could facilitate children’s voice as they are capable of their own opinions and in expressing wishes; that wishes often include to be seen as normal and not to maintain a relationship with perpetrator; could reveal ongoing safety concerns and the need for stability and continuity. Furthermore, recognition of varied responses, within sibling groups and wider families is essential.

In summary, we know little about children’s experiences and needs pre-homicide in the studies nor about their life circumstances and recovery process in years following (Alisic, Groot, Snetselaar, Stroeken, Hehenkamp, & van de Putte, 2017; Alisic, Groot, Snetselaar, Stroeken, & van de Putte, 2017; Mertin, 2019); reviews suggest further attention is needed on children’s ongoing and changing needs over time, relationships between different family members (including caregivers) and needs across services (Mertin, 2019). This informs the socioecological life course and multi-informant approach of our study.

### The UK and Ireland Context

The UK and Ireland context for this study is one in which there has been increased policy, practice and academic focus on IPH, yet continuing invisibility of children experiencing domestic abuse, even in circumstances of IPH (Stanley et al., 2019). The introduction of domestic homicide review (DHR) in parts of the UK, and internationally, allows for investigation of the circumstances surrounding the death of a person resulting from domestic abuse and learning surrounding IPH. Analysis of DHRs England and Wales since 2011 are helpful in informing practice, policy and academic approaches. Whilst DHRs provide a multiagency perspective there remains scant focus on children, even though children’s involvement in IPH includes witnessing the homicide and/or the outcome, and calling for help (Stanley et al., 2019) We have little data on the children involved: the age, sex and ethnicity of children is not systematically recorded (Chantler et al., 2023); children themselves are not commonly involved in the DHR leading to partial accounts (Stanley et al., 2019) and DHRs include very little information on children’s experiences and the practical and emotional consequences for them (Stanley et al., 2019). A DHR’s objective is to identify lessons from the death concerning the effectiveness of local professionals and organizations in safeguarding victims, both independently and collaboratively, but at present the child co-victim is largely neglected. Stanley et al. (2019) recognize DHR accounts as constructed and partial, particularly given the lack of involvement in the review process of bereaved children and they suggest DHRs widen the angle of vision to include children, produce more authentic narratives and, vitally, improve capacity to focus on future care of survivors and recovery of children. This study is the first research in the UK to center children’s life course narratives of bereavement due to parental IPH and focus on the health and wellbeing of these children in the immediate aftermath of the death and longer-term.

## Theoretical Background

A life course approach focuses on exploring human development within an ecological context (Elder & Rockwell, 1979). The origins of the socio-ecological model are similar to this in that it started out as a conceptual model to understand human development in the 1970s and was later formalized into a theory in the 1980s by Urie Bronfenbrenner (Kilanowski, 2017). The socio-ecological model considers the complex interplay between individual, relationship, community, and societal factors (Bronfenbrenner, 1979). A key concept of the socio-ecological model is the notion of proximal processes, which recognizes the continuous interactions between an individual and their environment across time (Merçon-Vargas et al., 2020). Bronfenbrenner’s socio-ecological model fundamentally allows for a holistic approach to understanding the various dimensions to human development while acknowledging the dynamic interplay between each of these (Bronfenbrenner, 1977). Development implies change over time and there is consensus on what is considered normative, structured and orderly change. Some change however, is chaotic, irregular and unpredictable and contributes to the decline as opposed to growth at the individual level (Bengtson & Allen, 1993).

Our study is based on Bronfenbrenner’s later development of bioecological theory (1993-2006) where proximal processes are the primary mechanism used to define and understand interactions between a person and their environment (Rosa & Tudge, 2013). More specifically, we will draw on the Process-Person-Context-Time model to discuss our findings; the model translates to unravelling the proximal and distal processes resulting from a set of personal experiences and circumstances and their effect on aspects of life over time (Bronfenbrenner & Morris, 2006; Rosa & Tudge, 2013).

The circumstances in which CYP bereaved by parental IPH find themselves are nuanced and interconnected. Understanding how these factors confluence in the aftermath of bereavement will help shed light on the effects of personal characteristics such as age, sex and disability status; with wider contextual factors, such as other life events, education and employment, socioeconomic circumstances, and the effect of time on both short- and long-term needs and outcomes. To address the gap in existing knowledge, we have applied a socio-ecological model to the themes arising from our research, which sits within a life course framework. We are keen to identify pivotal points on the exposure to DVA and IPH and how it relates to a CYP’s development both in the immediate aftermath and across the life course.

We combine the life course socio-ecological approach with childhood theory to recognize the knowledge and competence of the (remembered) voice of the child; acknowledge children as social actors who are experts in their own lives (James et al., 1998) who exercise relational and intergenerational agency (see Sutterlüty & Tisdall, 2019) contribute and change circumstances for themselves, families and others (Abebe & Ofosu-Kusi, 2016), as well as being potential agents of change in communities, research, practice and policy (Houghton, 2018; Malone & Hartung, 2010). The study’s approach reflects the earlier paradigm shift in wider domestic abuse literature to center children’s perspectives on experiences and help-seeking as key actors and informants (Mullender et al., 2002), recognize agency, strengths, relationship management, key transitions and turning points, impacts and hopes (Arai et al., 2021) and ensure their inclusion in domestic abuse narratives (Morris, 2009); an approach largely unrealized in the partial and mainly proxy accounts of children’s experiences of IPH so far. The study’s life course approach necessitates an adult lens of (their own) childhood though centers participants’ (retrospective) constructions of their childhood social worlds (Danby, 2009) and, unusually, allows for exploration of reconstructions and socio-ecological impacts across time.

Whilst the dearth of child and survivor-centered studies in this field may be indicative of protectionist concerns of re-traumatization; researchers and young survivors themselves have argued for inclusion of children in research as experts (Houghton, 2015; Øverlien & Holt, 2019); the worst certainly has happened for children bereaved by IPH (paraphrasing Øverlien & Holt, 2019). To put children at the center of safety [and recovery], Warrington and Larkins (2019) argue that we must challenge the false juxtaposition of protection and participation rights (UNCRC) and promote survivors’ rights to participation as a necessary component of individual and collective protection. Central to this debate is the role of children in decision-making on provision and allocation of resources to address needs and outcomes of those experiencing abuse (Houghton, 2018; Warrington & Larkins, 2019): a key aspect of this study is to ensure ‘the view of the child must be taken into account to determine the best interests of the child’ (UNCRC, General Comment 12, paragraph 53) when the child is bereaved due to IPH.

## Methods

### Setting

This study draws on data from a 4-year research project exploring experiences of home, relationships and identity among CYP bereaved by IPH in the UK, Ireland and Australia. This paper draws upon data from the UK and Ireland, the focus of this analysis was on the experiences of children and young people in the UK & Ireland in order to relate it to, and inform, the currently evolving policies in this area. The main aim of the research project was to gain the perspectives of those with experience of IPH either through lived experience as a child; a caregiver for a child bereaved in this way; and as a professional whose work involved children post parental IPH. The design of the overall research project was developed in collaboration with people with lived experience of parental IPH, one being a co-investigator and one as an advisor to the project.

The aim of this study is to *position the needs of CYP immediately after parental bereavement due to IPH and across the life course within a 5 dimensional temporal socio-ecological model* (Bronfenbrenner & Morris, 2006; Center for Substance Abuse Treatment (US), 2014). Our main research questions are:

- What are the themes related to children’s experiences of bereavement due to intimate partner homicide and under which dimension/s of the socio-ecological model do our themes fall?
- What circumstantial factors contribute to noticeably different life trajectories for CYP bereaved by parental IPH in the UK & Ireland?

We will then discuss who should be responsible for targeting interventions and change at those level/s to avert and remediate the negative consequences of the experience.

### Design & Recruitment

The study design is qualitative and draws on 29 in-depth interviews with 32 participants across three main informant categories: those with lived experience of parental IPH; current or former caregivers to those with lived experience of parental IPH; and professionals with experience of working with individuals who have experienced parental IPH.

#### Inclusion Criteria for Participation

Participants needed to meet the following inclusion criteria to participate. For all three informant categories, participants needed to be living/working in the UK or Ireland. For those with lived experience they must have been under the age of 18 when they were bereaved by IPH, be 12 years or older at the time of the interview and be at least 6 months post-homicide/suicide. Caregivers could be current or previous caregivers, with the definition including foster carers, family members and family friends who have spent a significant amount of time with the child or young person. Allowing flexibility about who qualifies as a caregiver ensured we did not alienate or exclude potential participants due to cultural variations in arrangements. One caregiver included in our sample deviated slightly from the inclusion criteria set out here. The caregiver interviewed was the sister of a man who died by suicide in the context of DVA and was pregnant at the time of her brother’s death. Her struggle after this event greatly impacted her relationship with her new baby and was included to record the effects IPH has on CYP within a wider context. As for professionals, qualifying was based on direct professional experience of working with children and families who have experienced IPH, ideally within the last 3 years and in any capacity. Capacity includes all relevant services such as children’s social work services, youth services, victim support organizations, health providers, educationalists, and police officers.

Our definition of IPH naturally includes cases of homicide involving current or former intimate partners, in addition to intimate partner violence (IPV) related suicides. Including cases where a person has died by suicide and there was a clear link to IPV prior to their death, is important to begin to understand the different ways in which IPV can manifest, leading to loss of life and consequently affecting CYP (Rowlands & Dangar, 2023).

#### Recruitment

Participants were recruited through three main strategies. These followed the same protocol whereby details of the study were disseminated either directly to potential participants or, on a broader scale through established networks, and individuals who expressed potential interest in taking part were invited to contact the research team directly.

Our first strategy with regard to recruiting individuals with lived experience and caregivers involved collaborating with two key organizations within the UK who specialize in supporting children and families bereaved by IPH. These organizations are Assist Trauma Care, and Advocacy After Fatal Domestic Abuse (AAFDA). Both organizations took our inclusion criteria into account when assessing reaching out to individuals they have supported and acting as a first point of contact to establish whether potentials participants were happy to be approached by the research team with more information about participation. Letters were mailed to each potential participant - individuals with lived experience or carers. This was followed up by a phone call from the research team to check if a participant had received the letter and gauge their interest in participating. Just under half of our final sample (44%) was recruited in this way.

The remaining 56% of our sample was recruited by personal contacts, disseminating information about the study through various established networks such as, Commissioners for CYP, victim support services, clinical services, and professional associations throughout the UK and Ireland. We also asked all participants who were interviewed whether they were aware of any other potential participants. When a potential participant was identified, we asked participants to share information about the study with their contacts accordingly and a member of the research team followed up with them. Participants falling into the lived experience and caregiver category received a £15 gift card each to acknowledge their participation - professionals did not receive a gift card.

#### Ethics

The project was approved by the Medicine & Dentistry Human Ethics Sub- Committee at the University of Melbourne and the University of Edinburgh's School of Social and Political Science Research Ethics Committee in November 2020 and September 2021, respectively. This provided approval for interviews in the UK and Ireland in line with the General Data Protection Regulation (EU) 2016/679 that applies to both jurisdictions. We obtained informed consent from all study participants, whereby participants were fully aware of the study’s purpose, procedures, risks, and benefits before agreeing to participate. Most consent was completed online and recorded through Qualtrics XM. In other circumstances where this option was not accessible to some participants, oral consent was recorded by the interviewer before recording and starting the interview.

Participants had the option to withdraw at any time during and after participating in the research project, however, it was made clear that already published analyses that used data from ‘withdrawn consent’ cannot be redacted. The privacy and confidentiality of participants was guaranteed by protecting their identities and ensuring that their personal information and data are kept secure. At time of publication no interviewees had withdrawn consent to their participation in the study.

### Data Collection

All interviews were carried out by one of two researchers, one with a social work background and 35 years of experience working with children and families in the field of family violence. The second researcher has a public health and development background with 16 years of experience working in child protection, some of which are with children and families from marginalized populations and refugees of war. Basic demographic data was collected during the online consent process and researchers recorded links between participants with LE, caregivers to those with LE, and professionals. We ended up with three dyads and two triads within our sample (i.e. more than one individual who was connected to the same homicide – such as a young person and their carer). These interviews allowed for a more holistic understanding of the experiences and needs of CYP recently bereaved by parental IPH, taking into account the varying perspectives and unmet needs of both the CYP but also their care givers (often bereaved grandparents, aunts and uncles) and professionals dealing with these cases. The research interviews commenced during the second year of the pandemic, and interviewees were given the option of being interviewed in person or online. Most interviews took place online with the exception of two which took place in person.

#### Safeguarding Procedure

The study safeguarding protocol was led by the principle of Psychological First Aid (PFA) (Shultz & Forbes, 2014). PFA is a method of providing immediate emotional and psychological support to individuals who have experienced a traumatic event, crisis, or overwhelming stress. It aims to reduce the initial distress and foster resilience by addressing the emotional and psychological needs of those affected. In our study we endeavored to use the principles of safety and comfort, and contact and connection. This was carried out by safeguarding participants’ emotionally through listening actively and offering reassurance to ensure they felt heard and understood. A debriefing paper with a list of available counselling and mental health services for participants to access was shared during consent and at the end of the interview. Interviewers also checked-in and followed up with participants two days and two weeks post interview and discussed any needs with them. Among the interview team, regular debriefing and reflection sessions were carried out during the period of data collection. These sessions allowed for emotional support, reflection and processing in addition to vicarious trauma prevention.

### Data Analysis

Incorporating life course theory to the application of a socio-ecological model on our data ensures that temporality of lived experience is captured and reflected. Time is distinguished in three different ways within life course theory: individual time, generational time and historical time (Price et al., 2000). Similarly Bronfenbrenner and Morris describe three levels of time: microtime, mesotime and macrotime (Rosa & Tudge, 2013). In relation to ongoing proximal processes, microtime represents “continuity” versus “discontinuity”, mesotime represents the frequency in which these episodes occur over days and weeks, and macrotime “focuses on the changing expectations and events in the larger society, both within and across generations” (Bronfenbrenner & Morris, 2006, p. 796; Rosa & Tudge, 2013). To ensure a holistic understanding of the experiences of CYP bereaved by parental IPH, it is important to transcend microtime and mesotime to investigate the evolving narrative of close bonds within families against the societal backdrop of these enduring relationships, considering their connection to social organizational and historical context (macrotime/historic time) (Bengtson & Allen, 1993).

Temporality not only allows us to capture the duration of an event but inherently implies change and transitions. This underlying theoretical perspective helps us capture the intersections between temporal relationships and perspectives within cultural and societal contexts. It is important to acknowledge that CYP’s experiences of DVA and subsequently bereavement due to parental IPH as traumatic events with lingering consequences. We are interested in establishing the key factors affecting proximal processes between the various levels of Bronfenbrenner’s socio-ecological model. These indicators will assist us in mapping preliminary transitional points alongside the short and long-term change in trajectory after bereavement. We incorporated our chrono-sphere (time) dimension as the fifth level of the socio-ecological model and recorded it horizontally rather than cyclically. Our main interest is to map the ways in which CYP navigate their developmental journeys by exploring trajectories from key transitional periods after bereavement. Developmental journeys for the context of this study were broadly defined to encompass the home and living situation, identity development and the development of relationships with family and friends after being bereaved.

Proximal processes as described by Bronfenbrenner implies “almost always acting in a positive way on developmental outcomes” (Rosa & Tudge, 2013, p. 252), either through the promotion of competence or the reduction of dysfunctional outcomes (Merçon-Vargas et al., 2020). Bronfenbrenner also states that higher levels of proximal processes lead to lower levels of dysfunction (Bronfenbrenner & Morris, 1998). An extension to this concept was proposed by Merçon-Vargas et al. (2020), that describes what they term *inverse proximal processes,* which primarily comprises detrimental interactions, exhibiting a reversed correlation with the developmental outcome compared to Bronfenbrenner’s proposed relationship. Whereby, “higher levels of inverse proximal processes would lead to greater dysfunctional outcomes”, similarly this connection would exhibit greater strength in less advantaged environments (Merçon-Vargas et al., 2020, p. 331). Proximal processes in our study are bi-directional and can either promote or hinder well-being.

We undertook a reflexive thematic analysis approach to the rich data emerging from our interviews and followed the five phases of doing a thematic analysis as set by (Braun & Clarke, 2006). The UK and Ireland research team met on a regular basis to discuss emerging themes during the data collection part of the project and after data collection was completed. Thematic analysis and initial coding was undertaken by the first author who read and checked all interview transcripts, this process was inductive and ‘data-driven’ to ensure the thematic analysis was centering the voice of children (Braun & Clarke, 2006; James et al., 1998). During the thematic coding process, codes were extracted into an excel sheet, the emerging themes were discussed during research team meetings. This allowed for sharing, reviewing and reflecting on the arising themes and for the extracted codes to be discussed and refined by the entire research team. Following this stage and after coding was completed, the final codes were subsequently mapped onto the five dimensions of the socio-ecological model: individual; interpersonal; community; society and period in time (Bronfenbrenner & Morris, 1998; Center for Substance Abuse Treatment (US), 2014). For period in time, codes focused on aspects of micro-, meso-, and macro-time.

## Results

A summary of the 32 participants interviewed across all three informant types can be seen in Tables 1, 2, and 3. We conducted 10 interviews with individuals with LE, with an age range of 17-52; eight of these were aged 17-28. A total of nine interviews were conducted with 12 carers, and 10 interviews with professionals. Drawing on the entire sample of interviewees, an additional 23 individual cases of CYP bereaved before the age of 18 were counted and analyzed. In all of the 10 interviews of those with LE, the lost parent was the mother.

**Table 1:** Characteristics of Lived experience sample (*n* = 10)

Gender	Age at bereavement (years)	Age at interview (years)	Number of years between bereavement and interview	Parent murdered/died by suicide	Perpetrator type	Sex of perpetrator
Female	14	28	14	Mother	Former/current partner	Male
Male	7	17	10	Mother	Other parent	Male
Female	15	25	10	Mother	Former/current partner	Male
Female	9	18	9	Mother	Former/current partner	Male
Non-binary	17	23	6	Mother	Former/current partner	Male
Female	10	28	18	Mother	Other parent	Male
Male	2	20	18	Mother	Other parent	Male
Female	11	23	12	Mother	Other parent	Male
Female	13	52	39	Mother	Other parent	Male
Female	.5	49	48.5	Mother	Other parent	Male

**Table 2:** Characteristics of carer sample (*n* = 12)

Gender	Age child/ren at bereavement (years)	Relationship to child/ren bereaved by IPH	Parent murdered/died by suicide	Perpetrator type	Sex of perpetrator
Female & Male	13 &15	Foster carers	Mother	Other parent	Male
Female	6 & 8	Foster carer	Mother	Other parent	Male
Female & Male	6 & 8	Aunt & Uncle	Mother	Other parent	Male
Female & Male	9	Grandparents	Mother	Former/current partner	Male
Female	12 &13	Grandmother	Mother	Former/current partner	Male
Female	0.1	Mother	Maternal uncle	Former/current partner	Female
Female	7 & 9	Grandmother	Mother	Other parent	Male
Female	2.5, 7 & 10	Maternal Aunt	Mother	Other parent	Male
Male	16	Distant relative	Father	Other parent	Female

**Table 3:** Characteristics of professionals’ sample (*n* = 10)

Gender	Role	Number of DH cases worked
Female	Independent Chair - Domestic Homicide Reviews	10
Female	Independent Chair - Domestic Homicide Reviews	
Female	CEO / DHR Chair	2
Male	Domestic Homicide Review Chair	18
Male	Associate DHR Chair	10
Male	Law enforcement Specialist unit VAW	
Female	Consultant Community Pediatrician	1
Female	Key Support Worker	3
Male	Police Officer	1
Female	Support Worker/Counsellor	1

Table 4 summarizes our wider sample of the 23 additional CYP with LE which was drawn from the individual cases discussed across all interviews. The total number of 33 individuals is based on the 10 interviews with those with LE and the additional 23 cases discussed across all interviewees and extracted.

**Table 4:** Characteristics of extracted LE sample cases discussed across interviews (*n* = 23)

Gender	Age at bereavement (years)	Parent murdered	Perpetrator type	Sex of perpetrator
Male	7	Mother	Other parent	Male
Female	16	Father	Other parent	Male
Male	7	Mother	Other parent	Male
Female	6	Mother	Former/current partner	Male
Female	7	Mother	Other parent	Female
Male	13	Father	Former/current partner	Male
Male	7	Mother	Other parent	Male
Female	11	Mother	Other parent	Male
Male	3	Mother	Former/current partner	Male
Male	4	Mother	Former/current partner	Male
Female	8	Mother	Former/current partner	Male
Male	10	Mother	Former/current partner	Male
Female	1.5	Mother	Former/current partner	Male
Male	5	Mother	Former/current partner	Female
Male	-4^a^	Uncle	Other parent	Male
Female	12	Mother	Other parent	Male
Male	13	Mother	Other parent	Male
Male	8	Mother	Other parent	Male
Male	6	Mother	Former/current partner	Male
Female	8	Mother	Former/current partner	Male
Female	6	Mother	Other parent	Male
Male	13	Mother	Other parent	Male
Male	16	Mother	Other parent	Male

^a^This case involved the maternal uncle of the unborn child and the interview explored the challenges experienced by the new mother and how it affected her bonding with her baby

Our complete sample shows a representation of bereavement across every age between 0 and 18 years. The mean age at bereavement due to parental IPH among our sample of LE interviewees (*N* = 10) is *M = 9.9 years, SD = 4.8.* The mean age at bereavement due to parental IPH among our complete sample of LE across all interviews (*N* = 33) is *M = 8.5 years, SD = 5.3.* We will present the results by answering our two main research questions.

### What Are the Themes Related to Children’s Experiences of Bereavement Due to Intimate Partner Homicide and under which Dimension/s of the Socio-Ecological Model Do our Themes Fall?

In this section, we will discuss which level of the socio-ecological model our main themes and sub-themes fit. Due to the nature of the fifth dimension ‘period in time’, this will be discussed within each of the remaining four dimensions and the implications of time on necessary support.

### Individual

Exploring the needs of CYP from the individual level first helps provide a basis for the myriad connections that transcend into other socio-ecological dimensions such as relationships, community and society at large. Starting with the individual in our model, allowed us to stay focused on CYP and their position in both time and societal space in the aftermath of bereavement.

#### Identity and Life Narrative

Identity and life story start to grow in childhood; early adolescence is a critical period when identity development becomes especially pronounced and takes on added significance within our sample of participants with LE. The majority of interviewed participants with LE describe the effects of bereavement on their sense of self as something that started in early to late adolescence. There are distinct differences in the way identity development is impacted based on whether the perpetrator is their other biological parent as opposed to a current/former partner of the deceased parent. Participants of all genders whose father killed their mother shared how they were/are reluctant to enter into relationships for fear of being like their perpetrator parent. These inverse proximal processes affected CYP mostly during adolescence and early adulthood. On the level of microtime the experience was continuous and at the mesotime level they lasted for years.

about 15/16… *I was scared to go into relationship* with a female *because I did think that I would do what my dad did*. Growing up, I did think like that. *So that's why I was fearful of getting into relationship*. (Male with LE, 20, UK)

Participants from a mixed-heritage background clung onto and identified more with one of the ethnic identities. In a sibling dyad from our sample, the older female sibling who was 10, at the time of bereavement, describes how she embraced the deceased mother’s ethnic identity and culture. The younger sibling who was under three years at the time of bereavement and did not have any conscious memories of their parents, was attracted to the perpetrator’s ethnic identity, in so much, as to search for understanding of that side of them, that they did not know or have access to growing up:

So I do think [for my younger brother] it was more of a cultural identity thing (…) all my friends at school were white, and I was the only black one (…) whereas [my younger brother] grew up, and went to the same school, and he had a big group of friends, all of his friends black (…) I think he's really struggled with kind of growing up in like a white family and how he feels like an outcast (Female with LE, 28, UK)

#### Non-normalcy, Alienation and Isolation

The lack of stability in CYPs’ lives following bereavement contributed towards feelings of non-normalcy, both in the immediate aftermath and across the life course. Participants often felt alienated due to changes in living arrangements, sudden change in school, and bullying from other children at school and within the community generally. Support networks dictated much about the CYP’s immediate experience in the aftermath of IPH, however stigma was experienced despite this and notoriety within the community was inevitable. This inverse proximal process was experienced as continuous on the level of microtime and at the mesotime level was long-lasting. For most of our sample these feelings were life-long:

…I was very uncertain a lot of time. Because we assumed to be someplace one time someplace another time I was thinking, this is how it’s gonna be for the whole of my life*. Am I just gonna be in some perpetual state of motion*, it just created a lot of uncertainty… *was a bit of an odd kid. Definitely a very odd kid. So still am arguably.* (Male with LE, 17, UK)

Some described feeling as if they were carrying a big secret whereby no one could really know them or understand their experience. Participants described how they have to constantly confront their past during new encounters with people at various stages of their lives, to explain why they didn’t have a mother and/or father: *“…*it feels very isolating. I feel like I’m walking round with this like elephant behind me that no one knows about… people don’t really know the whole me*”* (Female with LE, 28, UK)*.* These ongoing confrontations affected how participants relate to others, both on a mundane level and within intimate relationships and friendships:

I tell people that it’s a bit like maybe in the 1970s or 80s. If you were gay, coming out, it’s like an experience of coming out when you tell somebody about this. And it would be really great if that wasn’t how it was. *Because it’s like you’re living with a horrific story, secret. And so, no one can ever really know you.* Because you’ve always got this thing that you’re hiding. And that’s awful because… *I’m not a bad person. I didn’t kill anybody but … I feel like it. (*Female with LE, 49, UK)

Data from and about those with LE in our sample touches on the feeling of ‘otherness’ experienced by CYP post bereavement, where friends, acquaintances, teachers and others struggle to speak to them about what had happened. Participants seemed to understand that others are upset about what has happened, and are likely worried about upsetting the child. However, this can be experienced as others withdrawing from the child, and a sense that they are ‘different’ after the incident. This resulted in feeling silenced where those with LE had nowhere to process their experience:

*…we don't really speak about it in school, do we?* We don't speak about it even in colleges, unless you're in the social work department. And even then, it's a big topic. Do you know, nobody wants to think of the worst thing in the world and truthfully, *being a martyr is the worst thing in the world*. Unless you're taking rape into consideration, *the worst thing that you can possibly experience is murder*. (Non-binary person with LE, 23, UK)

Both participants with LE and their carers mentioned the benefit of knowing and talking to others who have been through a similar bereavement. This helped participants relate to others and recognize that they are not the only ones who have gone through such an horrific experience. Residential weekends were offered by the charity Winston’s Wish in the UK, which brought together children bereaved through similar circumstances (homicide). For the majority of our sample however, they were never able to talk about the loss of their parent and meet others with similar life experience. This intensified the effect of the homicide/suicide on their sense of self and their perceptions of others not ever really knowing them.

Identity development is one of the key developmental tasks during adolescence (Branje et al., 2021; Erikson, 1959). Personal identity is also closely linked to social identity and is informed and shaped by social relationships and social contexts that pertain to an individual. Drawing on the proximal and distal processes that shape these developmental journeys, our data suggests that the cognitive age of a CYP at the time of bereavement, among other contextual factors, can potentially shed light on ways the event may affect their sense of identity and the age-appropriate responses and interventions that can be offered. Wider literature informs us that children bereaved during key developmental transitions are at higher risk of detrimental trajectories if these exposures are not addressed appropriately in the immediate aftermath of bereavement (Osterweis et al., 1984; Pham et al., 2018); which seemed particularly acute in IPH circumstances. Aligning with bereavement and identity literature, our findings also indicate that to mitigate identity based tensions, guilt and feelings of non-normalcy, there was a clear need for support and services that promote emotional-regulation in order to maintain continuity for bereaved CYP’s sense of self (Alvis et al., 2022; Berzonsky, 2011).

#### Resilience

For the majority of our sample, especially those who did not witness the murder, there was an acknowledged resilience in how they processed their experience and used it as a source of strength. Participants described how they matured very quickly and felt like ‘they had to’. This transition highlights the immediate switch CYP recently bereaved by IPH go through as their world is turned upside down. This finding is a good example of how proximal processes can be bi-directional, and when adversity befalls an individual, they are able to draw on individual agentic resources to overcome the adversity, despite the greatly disadvantaged position they find themselves in. In terms of time, at the microtime level, there is an immediate discontinuity to the exposure of DVA which with time allows the CYP to draw on these resources to overcome the experience.

*I feel like I matured very quickly* because *I felt like I had to… I was gonna get on with things* but I definitely feel like *I matured very quickly.* (Female with LE, 18, UK)

*…*all I knew is *I wanted to be successful. That's all I had going for me at the time, I was just like, I'm going to get me head into school, I'm going to work really hard. I'm going to do this.* (Female with LE, 23, UK)

I went through lots of stuff it *has made me very strong minded. Therefore, it has made me ambitious as well.* By thinking that I can achieve anything I'll put behind myself and the perseverance to complete anything… *when I want to achieve something. I will make sure I will achieve it, because I'm strong minded. I'll persevere through it.* (Male with LE, 20, UK)

Participants with LE in our sample also expressed how they often felt alienated due to changes in living arrangement, sudden change in school and bullying from other children, both at school and within the community generally. Not all notoriety was bad, however, and for a small number of our participants, those CYP benefited from their deceased parents’ good reputation and standing in the community. These benefits extended to early work opportunities and help during transitions from school into the work force or college for a minority of our participants with LE. Community support in instances such as these can contribute to CYP’s wellbeing and sense of resilience during key transitions.

Our findings support bi-directional synergetic domains that fit within the dimensions of the socio-ecological model. Similar findings from a recent study exploring resilience among children and adolescents who have been victims of domestic violence, reported low levels of school and community engagement and support as risk factors to low levels of resiliency, whereas stability and the establishment of routines helped endorse resiliency in the study sample (Hildebrand et al., 2019). A lack of support from the immediate interpersonal level (family and friends) and community (school and neighborhood) had detrimental effects via inverse proximal and distal processes on CYP’s resilience and mental health in both the short- and long-term post bereavement.

### Interpersonal: Relationships – Family & Friends

#### Kinship Care

For CYP where other family members took on the caring responsibilities, there was an evident expectation by the State for them to shoulder the burden without any financial help. Additionally, surviving family members who did take on the caring responsibilities often grandparent/s, aunts and uncles, were typically never offered any support in how to deal with child bereavement, or grief counselling for their own loss. Challenges at this level highlight the interconnectedness of inverse proximal processes between CYP’s individual needs with the needs of their family. This finding emphasizes that the process and context of parental IPH has a rippling effect that goes beyond the individual level into the interpersonal level of the socio-ecological model. Our analysis shows that these challenges persist over long periods of time (years) and are not transient; with long lasting damage that endures for both CYP and their families.

No financial support, all [the sister with caring responsibilities] got for Child 1, Child 2 and Child 3 was child benefit… was it [support] there? no and in fact, when the children moved from our mum and dad's to [my sister with caring responsibilities] they were removed from the free school meals. (Female Caregiver, UK)

The complexity of these circumstances, was described by one participant as ‘causing a lot of family conflict’, and led many families to breaking point as their own grief was not dealt with in a holistic way, if at all. Additionally, disagreement among family members about the best decisions for the surviving CYP in the immediate and longer-term puts a further strain on families. For those where the perpetrator is the other parent, there is the added complexity of dealing with two sides of an extended family, who can be seen to ‘battle it out’, in order to control a certain narrative in a way that is more desirable to them. Additionally, in at least one circumstance, those with LE suffered from financial exploitation from assets and pensions by the family members whose care they were in.

Professional participants in our study agreed that there is a need to better understand how decisions are made in terms of living arrangements, specifically the suitability and ability of family members to cope with their own grief while supporting the bereaved CYP who end up in their care.

…there's just such a whole range of not just emotional issues to kind of process, but also a whole range of practicalities, you know, suddenly, you were, you know, all your plans for retirement have to be thrown out the window, now you've got kind of two kids under 10 living with you. And actually, you don't have a spare bedroom for them. (Professional, UK)

There is something about some kind of fuller assessment and training in terms of fuller assessment… yes, it's really important that children get to stay with their families. But what does that look like? (Professional, UK)

From our caregiver participants, one interviewee was the pregnant sister of a man who died by suicide in the context of IPV. The effects of this loss on the new mother and her baby led to difficulties during the actual birth and bonding afterwards:

it was really hard to look after my baby. And so, when I look back on it, now I see how like vulnerable he was… a little baby from being looked after. And that sort of bonding period and things and as it happened, and not had a very easy time with birth” (Female caregiver, UK)

#### Coping and Escapism

Within the cases of those with LE, differences are seen between siblings, especially related to biological sex and witnessing the murder. Engagement in risky behavior and substance misuse was most likely in early and late adolescence across gender. However, there was some indication that this was worse and continued into adulthood for boys. This is further compounded when the home environment is not stable and their experience becomes one of rejection, alienation and isolation.

…it transpired actually, that *he started drinking*, because *he felt he wasn't protecting his sister*. And she was being harmed by the foster care. So *he was trying to blot it out*. (Female Caregiver, UK)

[grandson’s name] got a criminal record for violence and drug taking and blah, blah (Female Caregiver, UK)

I smoke a lot of weed, if you must know, that's probably what keeps me on a numb one… it is probably what keeps me calm right now. (Non-binary person with LE, 23, UK)

Others noted how they have experienced rejection from their immediate family and friends for their parents’ choices, and have been tarnished by the actions of either or both the victim and perpetrator. Some CYP with LE were able to maintain existing friendships, this seemed dependent on their age at bereavement and particularly true for children who were ten years or older. However, many stated that they did not trust people easily, often not sharing the reality of their deceased/incarcerated parents so they did not have to explain or go through the life event over and over.

### Community

#### Foster Care

There was variance among analyzed cases as to the appropriateness, quality of care and support received by bereaved CYP with LE. Some of the placements with foster carers were culturally sensitive and allowed a deeper level of support due to foster carers understanding of the cultural background of the CYP placed in their care. In a case of bereaved siblings, the support that was provided to the foster carer didn’t reach the CYP it was intended for and one of the siblings reports that they were financially exploited:

[Foster carer] was an absolutely horrible woman, very mentally manipulative and sort of like, oh, how do I really, say? She didn't quite care for us at all. She took money from us and from like, our mother's pension, actually, that's the same with the previous household. They also took money from our mother's pension. (Male with LE, 17, UK)

They've been physically mentally and financially abused... The [girl] has a scar on her leg from being pushed. I mean, (the boy) has Aspergers he's been picked on for having learning disabilities being called ‘the fixer upper’ (Female Caregiver, UK)

Overall, placement with appropriate foster carers was up to the services making the decisions, which had significant consequences for the CYP’s future. Furthermore, one foster care placement discussed by a professional participant, focused on the placement offering a fresh start for the child, allowing them to flourish. Similar findings from a study of 77 care leavers in Australia found that for the majority of participants their time in the Australian out-of-home care system was a time of instability and insecurity, both in terms of housing and relationships (Natalier & Johnson, 2015).

#### School

Schools are considered an important environment for children because of the stability and support they potentially offer in addition to the learning that children gain. In our data, and across all respondent types, the school environment was overall ill equipped at offering appropriate support to these children. This was true for CYP continuing at the same school or starting at a new school due to relocation. In only one of our cases from the wider sample of those with LE, was the school engaging with other services and taking part in monthly meetings regarding the child’s welfare.

Then when we were in school, we didn't *neither of us could fit in* because everyone, you know when you're making friends, what does that do what happened like ......., and then *it's you live with your nanna? That's weird* (Female with LE, 23 UK)

Support from schools was scarce across our LE interviewees, either due to schools not having the resources or lacking expertise in dealing with CYP bereaved by IPH. Like other services in the aftermath of a bereavement, it was up to school management and the availability of resources if any effort was put in to help with the transition of CYP during the aftermath of bereavement.

… school did not offer or … did not mention counselling … I mean, this is one area where I (…) hope things are done better now (…) had meetings with my sort of form tutor. Maybe once a month, but I wouldn't really say that they did much, they just checked in. (Female with LE, 28, UK)

There was counsellors … I was offered it at school, but that was kind of it, *wasn't then spoke about ever again…* And *I feel like maybe if I was maybe pushed a little bit more at that young age, I probably wouldn't be dealing with the things that I am dealing with now*. So I think give them that option. And tell them the benefits of that. (Female with LE, 25, UK)

Other research has identified the potential support that schools can provide, and the importance of the teacher’s role in being a trusted adult who children can disclose gender-based violence to (Montserrat et al., 2022). Teacher training on prevention and intervention in cases of IPV and IPH would create a safe space for disclosure and sharing.

### Society

From our data, it is evident that CYP experience structural and systematic alienation, not only during the immediate aftermath of bereavement but across the life course. Stories of societal injustice followed CYP throughout their life, as a consequence of being related to a convicted murderer. This experience was true for one of our participants with LE who shared an experience where their sibling, who witnessed the murder of their mother by their father and experienced bereavement, did not receive any support and consequently ended up with criminal convictions. The criminal convictions of close relatives (the father and brother in this case) has prevented the participant with LE from pursuing certain careers by disqualifying them from positions within the police and civil service:

I (…) finally decided, (…) [to] try and get in the police (…). [I] got all the way past me interview, did everything great! Vetting was the next thing… and they rang me last week, ‘Yeah, we fear, your vetting has been rejected..’ I wonder why that is? It won't be anything I've done. (…) It's because [of] what my dad did… (…) It's difficult. (…), You work hard for something. And then because of someone else's actions, I can't do what I want as a career. (…) It's awful… (…) I don't have a relationship with my dad, I have a relationship with my brother. Yes, he's not made the wisest decisions, but all his decisions and actions he's made are due to watching my mum die. (Female with LE, 23, UK)

#### Children’s Voice Is Rarely Elicited

Data from across all respondent types was resounding in that perpetrator rights were valued over children’s rights. Children were often not given any opportunities to voice their preference about where they ended up living and with which family members. Within families who were more aware of the implications and repercussions of decisions made in the immediate aftermath about where CYP ended up living, there was retrospective acknowledgment that they never actually asked the CYP for their views. Despite the need for some stability after bereavement, our LE interviewees shared that they would have relished a new start, somewhere where they were not known:

I think I remember there being talks whether or not we would stay in [home town] or whether or not we would go to [city name] to live with my [youngest aunt]. I think at that point, she had two children… but I don't think I was like, involved isn't the right word. But I don't think, *I think everyone kind of sort of decided to keep us in* [home town] so that we wouldn't be sort of taken out of school and everything that we knew. (Female with LE, 28, UK)

Additionally, our interviews uncovered that, bereaved CYP have mostly been living with domestic violence and abuse before and during the IPH and lack/ed. the language to relay their experience in meaningful ways immediately after bereavement. They also face stigma from those around them who are also grieving about what they feel can and cannot be shared. The lack of acknowledgment of their victim-survivor status only compounds their voice leading to any sense of agency being completely lost.

Many CYP also stated that information around the incident was not shared with them and that they were left in the dark about what had happened or was happening. This did not change when CYP reached adulthood, where they found it extremely difficult to access any court proceedings or information from the police investigation. Some participants stated that this information is ‘held at ransom’ by organizations which require relatively large sums of money to process the requests (such as court transcripts of the criminal trial) or refusal to provide the information in the first place:

they make it quite hard to then get information later down the line… I was looking into getting the transcripts of the court and stuff, but they don't keep all of it on file. And then I think you have to pay a few hundred pounds to get the transcripts and things like that and I just don't think they make it very clear, very easy, later down the line. So that's just what I personally think anyway, but I just feel like whether they’re young or not, I feel like they should be given the option on what they know and what they don't know. (Female with LE, 25, UK)

#### State Support

Lack of any financial support for kinship care, meant even middle-class families were stretched to the limit and had to use their own savings and resources to provide for the additional child/ren in their care. The system offered no consideration of the fact that these families had taken on the care of their bereaved family members and assumed that they would find a way to support them financially. A large percentage of kinship care falls upon retired grandparent/s, some of whom are only in receipt of state pensions. From our analysis across all respondent types, this does not seem to have changed over time:

When I was struggling to cope with different problems he was having. And when I said something to them, they said, but we're not here for you, we're Children's Services. That is what I was told. (Female Caregiver, UK)

There is no official counselling or therapeutic support geared towards children and young people bereaved by IPH. When families try to source support for bereaved CYP through their own volition, they are often denied access to counselling while the investigation is ongoing. This experience was echoed across all three informant types, with CYP being seen by the criminal justice system as witnesses and not as victims of IPV in their own right:

…they weren't allowed counselling for a year… because the police said what if they need to be interviewed for the case. My niece is 10, middle child was seven. What are you gonna get from them that you can't get now? (Female Caregiver, UK)

Life trajectories are all very different, even and especially between siblings. All interviewees with LE spoke of the need for mental health care across the life course and some spoke of the difference it may have made if they had accessed support earlier.

### What Circumstantial Factors Contribute to Significantly Different Life Trajectories for CYP Bereaved by Parental IPH in the UK & Ireland?

Three key themes emerged from our analysis of the circumstances and experiences of CYP bereaved by parental IPH. These are: 1. Children’s presence at the time of the murder; 2. Relatedness to the perpetrator and relationships with the wider family, and 3. Biological relatedness to the perpetrator and the response from others. These three themes mainly relate to the individual and interpersonal levels of the socio-ecological model; however, they transcend into the community and society levels in terms of response and support from the wider community and state.

#### Children’s presence at the time of the murder (witnessing the murder)

Our complete sample of LE highlights that CYP *who witness the violent death of a parent* at the hands of the other parent or a former or current partner talk about the trauma of the incident in a more direct way. Losing a parent and the catastrophic after effects they must adapt to carrying this experience, adds a layer of complexity to treating and supporting survivors. In our sample, four CYP witnessed the murder of their mother, two of whom have special educational needs and disabilities (SEND). They shared similar feelings of regret at not saving their mother and not being able to intervene.

…well {my grandson} said *it was his fault his mum died. He couldn't save her. He firmly believes he should have been able to stop his dad,* he said “*I threw me phone and me lollipop at it him”*. His dad is over six foot tall. Yeah, he might be skinny. But, he's over six foot tall. And *we're talking about a 10 year old kid*. I mean, God, my daughter was only five foot three. {My grandson} was nowhere near her height at that age of course. So he's absolutely inconsolable. (Caregiver LE07, UK)

There were several references to SEND children bereaved by parental IPH from all three informant types, those with lived experience who discussed sibling/s with SEND, caregivers, and professionals. The analysis identified a recent disclosure made by a SEND young adult to their caregiver (made four months prior to the interview). This highlights that children with additional support needs can comprehend the incident and require specialist intervention to suit their individual needs. Below is a quote from a caregiver who describes her nephew with SEND disclosing parts of the murder he had witnessed, 12 years after losing his mother:

… {my nephew with SEND}, probably heard the thud of (My sister) falling out of the bed. And he said, he went out to the landing, and he said, he saw his *“daddy out on the landing… and he had blood on his hands. And I think I should have gone and saved my mummy”.* And I said, “What did you do?” and he said, “so I just hid under the bed.” Nobody knew that. I mean, that was, I kid you not, *that was probably about 12 years after (My sister) died*… And then all of a sudden, he'd just come out and said that and *carried that with him as well, really all these years*. So actually, that's a big lesson. Just because you've got a learning need. You've got rights. (Female Caregiver, UK)

Another professional relayed the experience of a SEND child and how they were able to communicate what they had witnessed with alarmingly uncensored detail:

…somebody with a learning disability is very, very, very different from that child with a normal IQ… if you ask him very clearly what happened, he will give you a blood and guts response to what happened, which meant he was able to give a very good recall to the police. But with that is the horror of it. (Professional, UK)

A health professional in our sample described the experiences of a child who not only witnessed the murder of their mother and baby sister but was also sexually abused and raped by the perpetrator alongside her mother and much younger sibling. Despite the harrowing experience, the professional respondent relayed how the child’s life has improved and that despite having endured and witnessed the most heinous of crimes, is thriving both in her new home life (in care) and at school. This improvement in home life and life experience post maternal bereavement was due to the collaboration and openness between the multi-disciplinary team working on this child’s case, who ensured the child’s needs are at the heart of their decision making.

#### Relatedness to Perpetrator (Parent Vs. Current or Former Partner)

The relationship of the perpetrator to the CYP was a definitive branching in their lived experience. Branching in our study is defined as a turning point from which a bereaved CYP’s life takes a drastically different pathway based on circumstantial and contextual factors. Primarily, those CYP who are bereaved by parental IPH at the hands of their other parent, experience a double dose of loss and grief, whereas often cases involving a current or former partner leave the CYP with a surviving parent who was not involved in the homicide/suicide.

The immediate experiences for CYP in the aftermath of bereavement can also differ. When the perpetrator is the other parent, experiences of DVA in the home are common and are more prolonged and continuous in nature. This long-standing exposure to DVA can be considered the norm for the CYP in the immediate aftermath of loss and is often the only example they have of an intimate relationship. In terms of inverse proximal processes at the microtime level, these experiences of DVA for this group of CYP are continuous in nature. At the mesotime level, it denotes long periods of exposure, with the likelihood of it spanning their entire lives up until they are bereaved. The grieving process is therefore more complex as the CYP have to contend with the trauma of loss, guilt and sadness on the one hand and anger, frustration, confusion and even hatred on the other:

…and if I had the same kind of interview at age 11, *I would have told you my home life was normal, I would have told you, you know, things that went on were totally fine.* Like, I wasn't worried or scared or anything else… *now looking back at it, it was absolutely horrific.* Female Lived experience, 23, UK

It appears that despite the conviction of the other parent for the murder/suicide, parental rights are maintained from prison regardless of how a child or young person feels about contact with the perpetrator. Analysis of our complete sample showed parental rights were upheld favoring them over the rights of the children, which led to numerous examples of re-traumatization and coercive control after IPH, a common experience post-separation for children experiencing DVA (Katz et al., 2020). Children were rarely consulted about what they wanted by social services or the justice system:

One of the things that come out of the courts was [Perpetrator] was allowed to have school photographs every single year, sent to him in prison. He was allowed to see a copy of all the school reports. I fought really hard that he shouldn't see the school report unless the children … at that stage had a voice. Child 1… had the capacity to say*, I don't want that and was absolutely appalled at the thought of it for her or her brothers.* I used to say… when it's school photographs day, don't send them in, *because there isn't a school photograph that can be sent*. (Female Caregiver, UK)

In the aftermath of a homicide, relationships within the extended family can become complex, with both the victim’s family and the perpetrator’s family each trying to control the narrative. This can lead to tensions between the perpetrator’s and victim’s family. These differing perspectives, narratives and tensions impact on CYP, leading participants to feel isolated, unsettled and confused.

In cases where the perpetrator is not the child’s other biological parent, our analysis shows that CYP often suffer an intense but short-lived exposure to DVA with the potential of being directly abused themselves. These experiences in terms of inverse proximal processes of DVA are experienced as intermittent at the microtime level whereby the exposure is not necessarily continuous, with periods of discontinued exposure to DVA or just the one fatal experience of DVA (the deceased parents’ last intimate relationship). They are often alienated from family members prior to the homicide/suicide and at the meso level of inverse proximal processes are more likely to experience periods of drastic change and trauma.

In the immediate aftermath however, these CYP can often experience an improvement in their living situation, as they are no longer living within the context of DVA, where some get an opportunity to reconnect with family members from whom they have been alienated, resulting in strengthening of familial relationships. The key factor here being that the perpetrator is not related to them and bereavement brings a positive change to their life. At the level of mesotime, this frames the inverse proximal process of their exposure to DVA with a more definitive beginning and end that does not linger in the same way that it would for those where the perpetrator is the other parent:

*...her foster carer was just incredible… both of them amazing couple*… she was *just welcomed into this house with open arms*. And it never felt like it was a foster care placement… sort of *straightaway being welcomed into another family*, which I think has really been a *good source of stability for her*. (Professional, UK)

In cases where the other parent takes on the caring responsibility for the CYP, the unexpected caring can cause initial tensions in the relationship between the CYP and surviving parent. This is often due to the relationship historically being based around short visits and the other parent (father) not being fully prepared to deal with caring responsibilities which is further compounded by having to deal with a grieving traumatized child. At the level of mesotime, within inverse proximal processes, bereavement brings with it a new set of challenging factors at the interpersonal level:

Once I lost my mum, then moved in with my dad, which was *a big change*, because I went from seeing him… *like maybe once a month to then having to live with him*, which *was a massive change*. For me… it wasn't all smooth sailing at all*.* (Female with Lived Experience, 25 UK)

#### Assumptions about Biological Relations

Among our participants, the question of being “biologically related” to the perpetrator had wide implications. In cases where it was the ‘other parent’ who committed the crime, there can be certain complexities to this seemingly straightforward relationship. Family members, both those with LE and carers of those with LE, expressed concern regarding surviving CYP bereaved by IPH with this unique set of circumstances. In both the cases referred to below, perpetrators hold full parental responsibilities for the child/ren of the murdered mother, are considered to be the legal parent and therefore have parental responsibilities. Social services were described as being oblivious to the underlying nature of this seemingly straightforward relationship and the potential repercussions:

There’s another can of worms thing here… the fact of the matter, with my nephews at least is [they were conceived by] IVF and a sperm donor, *they're not related to the person who's in jail, you know, charged with the murder of their mother*, this is a known risk factor. Children being in the house are not biologically related to the man that's in the house, it's a risk factor for the mother getting killed (…) the social workers don't want to know anything about that. He's as much a father in their eyes… he's legally on paper, the father (…) social workers want to simplify it, they don't want to know about circumstances, *they don't want to know about the biological thing.* (Male Caregiver, Ireland)

Some of my brothers, the two brothers… were sent back to my father when he came out of prison. Yes. *They weren't his children… he took them because the court said he could have some back… the treatment that they had from him and his wife was appalling.* They were only little boys. (Female with lived experience, 52 UK)

This finding represents an inverse proximal process that covers all dimensions of the socio-ecological model and highlights microtime where it is occurring continuously for the CYP due to their status. At the macrotime level, this represents changes in expectations by society at large whereby it is becoming more lenient over time regarding the complexity of biology and relatedness within the context of DVA and IPH.

### Circumstantial Factors Leading to Proximal Processes

Emphasis is placed on the role played by the child in their own development by means of proximal processes (Bronfenbrenner, 1986, 1995; Bronfenbrenner & Morris, 1998, 2006). Bronfenbrenner highlights that proximal processes have a symbiotic relationship to personal characteristics, and the context in which they occur across place and space in time (Bronfenbrenner & Morris, 2006; Rosa & Tudge, 2013). The three key themes identified in our study represent a branching in the life trajectory based on the context and aspects of micro and meso time, which further compounded the experience of bereavement itself. For those children and young people who were present and witnessed the murder/suicide, it was reported that the life trajectory was much more fraught, with early criminal engagement, drug and substance misuse, reporting poorer mental health and less adjustment in both home life and relationships. These inverse proximal processes seemed to endure over time and did not resolve themselves. This can be seen within our data when compared to a sibling who did not witness the murder and how they were able to cope. This finding highlights the relevance of contextual factors relating to the incident that affects personal responses by bereaved CYP.

Again, relationship to the perpetrator showed a clear branching in life experience, especially regarding home life and relationships with family and friends. This branching highlights the differences in challenges that ensued post bereavement by those related to the perpetrator (being the other parent), as opposed to being unrelated (current or former partner). Post separation coercive control was widespread among CYP bereaved by parental IPH where the perpetrator was the other parent (such as demands for the child to visit them in prison). This finding is in keeping with research conducted in the UK and Finland exploring the mechanism in which coercive control continues to harm children post separation from fathers within the context of domestic abuse (Katz et al., 2020). Our findings about the added vulnerability of CYP who are not biologically related to the perpetrator echo those of previous studies (Radhakrishna et al., 2001) and suggest that within the context of CYP survivor-victims of parental IPH, consideration should be given to the potential of child maltreatment when they end up living in the same household with a non-biological perpetrator.

It is recognized that trauma in children with neurodevelopmental disorders is complex and occurs at higher lifetime prevalence rates than that in the general population (Hoover, 2020). Regardless of the type of additional support and/or disability present, simple transitions in everyday life are difficult during childhood for many SEND children (Gillan & Coughlan, 2010). Bereavement due to parental IPH further complicates these existing challenges due to the catastrophic event, in addition to the chronic or acute experience of domestic abuse. More acknowledgment and recognition are needed around the fact SEND children are aware of the traumatic experiences they witness and are able to hold onto those memories for long periods of time. Recent research on individuals over 18 years of age in residential care showed evidence of long lasting detrimental effects of potentially traumatic events (Berger et al., 2015). Our findings show similarly long-lasting effects from early childhood into adulthood. This finding highlights the interconnectedness between existing policies, service providers within the community and new caregivers (whether surviving family members or foster carers) and how awareness raising and tailored support for SEND children within the context of parental IPH is essential.

## Discussion

This study applies Bronfenbrenner’s socio-ecological model in its entirety based on a mode of discovery which he reiterated in many of his publications as the best way to reveal the interdependence between the various elements in the data (Bronfenbrenner, 1995, 2000; Bronfenbrenner & Morris, 2006; Rosa & Tudge, 2013). Applying the model in this way, highlighting proximal and distal processes as being either detrimental (inverse) or benevolent, allows for a much more inclusive and expansive use of the model. This study has sought a phenomenological understanding of the nested structures within the socio-ecological model highlighting the interconnectedness between both the distal and proximal bi-directional processes affecting CYP bereaved by parental IPH. However, when we discuss proximal processes in a bi-directional way, ambiguity persists regarding certain proximal processes due to their lack of clear-cut delineation.

Three key messages emerge from our analysis concerning children and young people’s experiences of bereavement due to parental IPH. Our first key message relates to the circumstantial factors pertaining to the homicide/suicide that are central and must be taken into account when responding in the immediate aftermath and across the life course. Three key themes were developed from our study which led to a branching in the life trajectory of bereaved CYP. These are; witnessing the murder; whether the perpetrator is the other parent or a current or former partner of the deceased, and, assumptions around biological relatedness. Identifying these in the immediate aftermath of a homicide could help guide practitioners and responders in the best way to support the bereaved child or young person. By acknowledging differences in circumstances, adapting responses accordingly and tailoring support in the immediate aftermath of bereavement, there are opportunities to ameliorate the potential negative consequences.

Secondly, the identification of appropriate caregivers and a holistic provision of support need to be embedded into guidance and legislation. Caregivers play a crucial part in shaping the life experience of bereaved CYP in the immediate aftermath of homicide, and across the life course. Taking on the role of a main caregiver and having to deal with grieving children of varying developmental ages is challenging. When caregivers happen to be family members or close family friends, they need support to deal with their own grief. In addition, financial support needs to be provided, possibly codified in law, to acknowledge the immense responsibility of taking on the care of bereaved CYP due to IPH.

Our third key message relates to necessary changes at both the community and societal levels. Changes in legislation are required to both support bereaved CYP due to parental IPH and to embed children’s rights within response mechanisms, ensuring they have a voice and are agents of change. We will discuss children’s rights and agency in the next section. Finally, we will explore the implications for policy and practice.

### Children’s Rights and Agency

Our study shows that children and young people’s rights are not adequately considered by public bodies in the UK and Ireland. Despite the UK being a signatory on the United Nations Convention on the Rights of the Child (UNCRC, 1989) since 1990 with it coming into force in January 1992 (The UK Government, 2010). Article 19 – the right to protection from violence, abuse and neglect – needs to be considered in relation to abuse before, during and after the IPH; recognizing children as victims of domestic abuse along a continuum, addressing the resultant individually expressed needs, that we learn may differ according to circumstance, biological relatedness, gender, culture, ability and age. The state is not taking appropriate measures to promote physical and psychological recovery (Article 39); children and young people were systematically let down by the lack of any guidance in legislation or policy on how to support child victims in the aftermath of bereavement due to parental IPH; noting this right to support must continue across the life course and at key junctures in their lives. Article 12 involves a child’s right to have a say and have views taken into account in decisions that affect their lives, which is missing across the life course, should include specific cognizance of children with a disability (Article 23) and recognition of culture (Article 30).Within our sample this resulted in parental (perpetrator) rights’ to make decisions about their children superseding children’s rights to have a say in who they lived with, and whether they had contact with their father or mother, including what information perpetrator’s received about and across their lives. Furthermore, children’s right to information (Article 17), particularly about the IPH, presents a serious gap at multiple levels across the life course, yet was key to their immediate and future well-being. There was no acknowledgment that CYP bereaved due to parental IPH are survivor-victims in their own right. Our data analysis shows that the experiences of the majority of bereaved CYP in our sample, including those with SEND, are in direct violation to a number of articles (stated above) within the UNCRC. These direct violations of the UNCRC represent the distal processes within the socio-ecological model that affect children through inverse proximal processes that permeate down from the societal and political level into the community and interpersonal levels. The lack of consideration and acknowledgment of these fundamental rights undermine the aspects of human agency available to CYP. Human agency is one of Elder’s four principles of life course development that has informed the later bioecological model developed by Bronfenbrenner and Morris where they assert that changes over time in the four dimensions of the socio-ecological model are not only products but also producers of historical change (Bronfenbrenner, 1995).

This study highlights that we are still some way from recognizing children as direct victims of domestic violence within the context of IPH. There is a clear struggle to encapsulate this in our law, policy, and as evidenced in our study, through practice responses. This status matters when we are looking at their ‘whole self’ and their experience of domestic violence and not ‘just’ their experience of bereavement. In order to ensure children’s rights are maintained and promoted, we recommend fostering children’s agency and listening to their voices to encourage and ensure a child-centric and child rights-based approach. This includes children’s rights being actively recognized and exercised in reality, and across the life course, as opposed to how these rights exist in theory but are not effectively upheld or accessible.

### Implications for Policy and Practice

Full consideration should be given to a relatively recent exemplar of ways to ensure responses are child centered and support is available and accessible across time is that of the orfani speciali (special orphan) in Italy; an approach that could address many aspects of our study. In January 2018, Italy became the first country in Europe to legislate support for ‘special orphans’ referring to the children of victims of femicide (Malizia, 2022). The Italian government pledged $13.5 million in funds the following year (Otte, 2020). The support is modelled holistically taking into account almost all aspects of the orphans’ lives; from assisting them through civil proceedings, provision of free therapy, support to continue education, start apprenticeships and/or enter the workforce. Additionally, the law protects the orfani speciali from financial exploitation by ensuring they receive their inheritance and preventing the perpetrator, from receiving a survivor’s pension. Uniquely, this progressive law also allows the orfani speciali to change their surname in order to disassociate themselves from the perpetrator (if related) to avoid notoriety (Otte, 2020).

On the third of October 2023, the Ministry of Justice through the UK Government announced the introduction of “Jade’s Law” as an amendment to the Victims and Prisoners Bill. This law automatically suspends parental rights for perpetrators of IPH *upon conviction* if they have parental responsibility for the children affected. The aim is to swiftly review these cases to ensure the best interests of the child, with an exemption in cases where victims of domestic abuse kill their abusers (Government Bill UK, 2023). The law is aimed at protecting children from post-separation coercive control by preventing convicted perpetrators from influencing key aspects of the children’s lives, such as accessing therapeutic support or changing schools. It is also expected to alleviate the burden on grieving individuals by eliminating the need to navigate the existing process of seeking restrictions on parental responsibility through family court proceedings, particularly during a challenging period (Ministry of Justice, 2023). This could address major concerns from this study; necessarily involve children’s participation to ascertain best interests and should be considered across jurisdictions.

However, despite this legislative progress, the law only comes into effect once a criminal conviction has been made. The investigation and criminal court process can last for many years, allowing access and parental rights for the incarcerated parent and continued limits to counselling in some cases. Additionally, comprehensive support for CYP who end up in “kinship care” remains a concern in the UK. Most local authorities do not adequately support family members who take on the care of children bereaved by parental IPH under kinship care arrangements. To address this gap, holistic support mechanisms for new caregivers, especially those placed with family members, need to be mobilized from key school staff and other relevant settings and services.

Our study supports the need for embedding immediate and lifelong access to therapeutic support for children bereaved by parental IPH within services (Alisic et al., 2015; Lewandowski et al., 2004; Mertin, 2019). This can be achieved by adopting a holistic approach to assessment and care planning, such as the example given by one of our professional participants (above), which highlights the importance of multi-agency cooperation and information sharing to ensure the best interests of the child are maintained and enacted upon. Our study also echoes others in highlighting the importance of providing much needed training and education for professionals likely to come into contact with CYP pre and post bereavement (Enander et al., 2024; Gomersall et al., 2024). Similar to other work done with CYP surviving bereavement (Alvis et al., 2022; Berzonsky, 2011), peer support was identified as beneficial and invaluable by our participants and we recommend incorporating peer support into service design to help mitigate feelings of non-normalcy, alienation and isolation. We also found that schools played an integral role for CYP post bereavement, acting as potential safe spaces. We recommend implementing school-based mental health programs, DVA informed, and developing integrated care pathways that incorporate mental health intervention into existing healthcare services such as primary care settings and schools (Enander et al., 2024; Steeves et al., 2011; Steeves & Parker, 2007).

In relation to DHRs, it seems recognition and inclusion of children’s individual narratives about previous domestic abuse and the IPH are crucial, and could form part of their immediate and ongoing recovery, as could the provision of information about IPH to children. There are potential benefits of DHRs looking at what happens to children in the immediate aftermath of a homicide/suicide. We support Stanley et al.’s (2019) call to widen involvement and include children as key actors and informants, and, importantly, include a focus on future care and recovery. Additionally, thought must be given to cases that do not qualify for a DHR due to limitations in the current scope across England, Wales and Northern Ireland. Scotland, is developing a Domestic Homicide and Suicide Model and has recently published results of an online consultation and targeted engagement with professionals working in the field of IPV and those with LE on the scope (Kurdi, 2023). Ireland does not have a DHR model set-up and could benefit from the lessons learnt from existing DHR models and Scotland’s exemplar process in setting one up. However, a recent study was published in Ireland exploring Familicide with recommendations addressing the set-up of a Domestic and Family Violence Death Review Model for Ireland (Department of Justice, 2023).

### Limitations

Participants were recruited through convenience sampling and word-of-mouth which could result in sampling bias, limited reach and limited diversity. However, we managed to achieve quite a diverse sample across the three categories of participants. Additionally, female participants outnumbered male participants in each of our informant categories, which means indications of gender differences need further exploration. There is potential for self-selection bias, as the recruitment model required participants to agree to be contacted before being approached by the research team or hearing about the study and getting in touch themselves. Another potential form of bias in our study is recall bias due to the retrospective recollection of life events by participants. No specific differences were found in relation to gender of perpetrator/victim in the wider sample and children’s needs, nor an opportunity to explore it with our LE participants due to our sample characteristics. Children’s experiences of bereavement due to IPH are multifaceted and complex, making it challenging to capture the intricacy of all aspects. Part of our sample includes multiple-informants talking about the same case/s which allowed us to triangulate information to deal with potential recall bias. Moreover, the application of the socio-ecological model to our sample has allowed us to navigate through these challenges by charting children’s experiences within the four temporal socio-ecological dimensions; individual, relationships, community and society.

## Conclusion

Children are often the invisible victims of IPH. While professionals and family members recognize the necessity of attending to children’s need to be cared for by an appropriate adult, there is relatively little acknowledgment of the wider range of important considerations relating to children’s requirements for support and recovery. Remembering in the process that we are dealing with children and adolescents, we should try to introduce some of the care free happiness children ideally experience growing up. In order to achieve this, we need to recognize that CYP’s experiences are part of a set of nested structures that interact in both positive and negative ways and depend on existing legislation, guidance and knowledge at every level within society.

Policy makers, legislators and response services can therefore use this information to tailor guidelines and interventions that provide practical guidance in the immediate aftermath of bereavement, while also providing for longer term support. Taking a holistic approach would ensure that there is a fit between societal and contextual challenges, together with individual agency capacities, to be enacted in these circumstances.

At present in the UK and Ireland there is no central or coordinated way of recording how many children are impacted by IPH. This has consequences for how we are able to plan for support, while taking into account the particular needs related to developmental age, sex, and ethnic background of CYP bereaved due to parental IPH. The authors recommend policy makers within the UK and Ireland embed laws and support services within a trauma informed socio-ecological model for CYP survivor-victims of parental IPH. By imbedding the needs of CYP within a socio-ecological model, it is hoped a roadmap will be dynamic, context specific and adaptable, evolving to address emerging challenges, new and intersecting inequalities.

## Data Availability

The participants of this study did not give written consent for their data to be shared publicly; due to the sensitive nature of the research supporting data is not available.
